# Leaving no-one behind: how CENP-E facilitates chromosome alignment

**DOI:** 10.1042/EBC20190073

**Published:** 2020-04-29

**Authors:** Benjamin Craske, Julie P.I. Welburn

**Affiliations:** Wellcome Trust Centre for Cell Biology, School of Biological Sciences, University of Edinburgh, Edinburgh EH9 3BF, Scotland, U.K.

**Keywords:** cellular reproduction, chromosome, kinetochore, microtubule motor, regulation

## Abstract

Chromosome alignment and biorientation is essential for mitotic progression and genomic stability. Most chromosomes align at the spindle equator in a motor-independent manner. However, a subset of polar kinetochores fail to bi-orient and require a microtubule motor-based transport mechanism to move to the cell equator. Centromere Protein E (CENP-E/KIF10) is a kinesin motor from the Kinesin-7 family, which localizes to unattached kinetochores during mitosis and utilizes plus-end directed microtubule motility to slide mono-oriented chromosomes to the spindle equator. Recent work has revealed how CENP-E cooperates with chromokinesins and dynein to mediate chromosome congression and highlighted its role at aligned chromosomes. Additionally, we have gained new mechanistic insights into the targeting and regulation of CENP-E motor activity at the kinetochore. Here, we will review the function of CENP-E in chromosome congression, the pathways that contribute to CENP-E loading at the kinetochore, and how CENP-E activity is regulated during mitosis.

## Introduction

The fidelity of chromosome segregation is critical for the maintenance of genomic stability and prevention of aneuploidy during cell division (reviewed in [1]). To ensure the equal distribution of the genome to daughter cells, the duplicated chromosomes are aligned and bioriented in the centre of the mitotic spindle before they are segregated. A macromolecular protein complex known as the kinetochore assembles on the centromere of sister chromatids and mediates their stable linkage to incoming spindle microtubules. The composition of the kinetochore is dynamically restructured throughout mitosis to (i) facilitate chromosome alignment and biorientation, (ii) sense, signal and correct erroneous kinetochore-microtubule attachments and (iii) mechanically couple chromosomes to the depolymerizing kinetochore fiber (K-fiber) microtubules during metaphase and anaphase. At centromeres, CENP-A-containing nucleosomes recruit CENP-C and the constitutively centromeric associated network of proteins (CCAN), which provide a structural link between chromatin and the core microtubule-binding hub of the outer kinetochore, known as the KMN (KNL1, Mis12 and Ndc80) network [2–8]. At the onset of mitosis, an expandable network known as the fibrous corona [9–11] assembles at the unattached outer kinetochore, stabilized by the oligomerization of Rod-Zw10-Zwilch (RZZ), and recruits Spindly, Mad1, Mad2 and CENP-E [12–14]. The ring- and crescent-shaped modules of the corona increase the surface area of kinetochores in prometaphase to promote kinetochore-microtubule attachments and accelerate chromosome congression [10,12,15], before compaction and disassembly in metaphase [16]. This microtubule search and capture pathway, driven by the dynamic properties of microtubules, promotes formation of bi-oriented attachments to spindle microtubules from opposite poles. Upon chromosome bi-orientation, kinetochores maintain connections to dynamic microtubule ends, which power chromosome movement through forces generated by microtubule depolymerization [17,18].

This microtubule-driven search and capture of kinetochores is a major mechanism of chromosome alignment (reviewed in [19]). However, chromosomes close to the spindle poles often only establish attachments to a single spindle pole and remain monotelic [20–22]. Mono-oriented chromosomes require a distinct pathway to successfully align at the equator [21]. This congression pathway is driven by microtubule motor-dependent forces, ultimately dominated by CENP-E [21,23–25]. In this review, we discuss the contribution of CENP-E towards motor-dependent chromosome congression, the recruitment pathways of CENP-E to the kinetochore and the regulation of CENP-E function in mitosis.

## Identification of CENP-E: a microtubule motor involved in chromosome alignment

**Figure 1:**
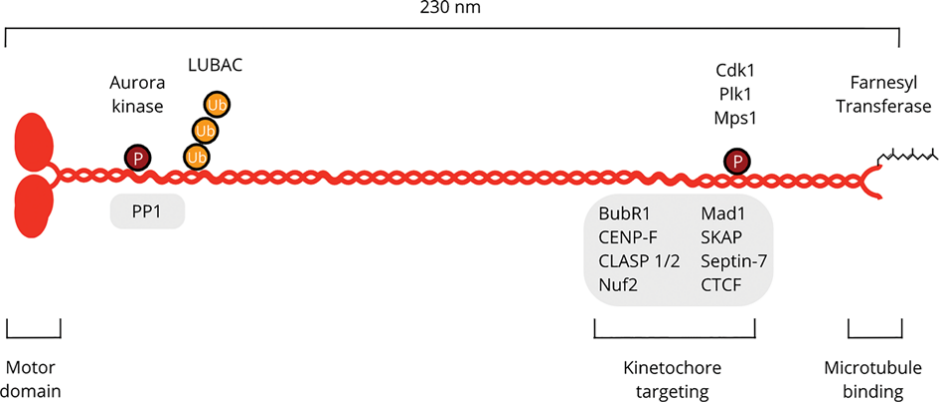
Schematic overview of CENP-E structure, modifications and known interactors The N terminus contains a globular motor domain, followed by an elongated stalk region consisting of discontinuous coiled-coils, which mediate dimerization. Phosphorylation (P), ubiquitination (Ub) and farnesylation regulate CENP-E activity and kinetochore localization. Several proteins have been identified that regulate CENP-E kinetochore recruitment (grey boxes).

CENP-E was established as a component of the outer kinetochore almost 30 years ago, shortly following the discovery of CENP-A, -B, -C and -D [26]. Subsequent work identified CENP-E as a kinetochore-bound motor, raising the idea that it may enable chromosome movement [27]. With a monomeric molecular weight of 316 kDa, CENP-E is the largest member of the kinesin superfamily [27]. CENP-E is a physiological homodimer comprising a N-terminal ATPase domain followed by an elongated stalk of discontinuous coiled-coils (Figure 1) [28–30]. Expression of CENP-E is up-regulated during G2 and peaks in M phase, before proteolytic degradation at mitotic exit [27,31]. During prometaphase, CENP-E is enriched at the crescents of the fibrous corona and decorates spindle microtubules [11,26]. CENP-E remains present at low levels at kinetochores in both metaphase and anaphase A [11,32], after disassembly of the corona by Dynein [16]. CENP-E relocalizes to the spindle midzone during anaphase B [11,26,32,33]. A function for CENP-E at the kinetochore was first highlighted following microinjection of polyclonal antibodies targeted against the CENP-E C terminus, which resulted in depletion of the motor from kinetochores and significantly amplified the occurrence of chromosome misalignments [34–36]. The authors also showed the kinesin motor domain was dispensable for kinetochore targeting and identified a minimal region within the C terminus required for CENP-E kinetochore localization [33–36].

## Mechanisms of CENP-E recruitment to kinetochores

Although several proteins have been implicated in kinetochore targeting of CENP-E, how it is specifically recruited to kinetochores remains under debate. BubR1 was initially identified as a CENP-E interactor during a Yeast Two-Hybrid (Y2H) screen, confirmed by almost stoichiometric coimmunoprecipitation of BubR1 from mitotic HeLa cells using anti-CENP-E antibodies [35,37]. BubR1 was first reported as a kinase involved in the Spindle Assembly Checkpoint (SAC) [36,38]. However, human BubR1 has more recently been re-classified as a pseudokinase, retaining a kinase-like domain that is catalytically inactive amongst vertebrate orthologues, with the exception of *Drosophila* BubR1 [39,40]. It associates with Bub3, Mad2 and Cdc20 to form the Mitotic Checkpoint Complex (MCC), which inhibits the Anaphase Promoting Complex (APC) [41,42]. BubR1 recruitment to the kinetochore temporally precedes CENP-E and depends on the mitotic kinase Bub1 [35]. A direct interaction between the C terminus of CENP-E and the pseudokinase domain of human BubR1 has been reconstituted *in vitro* [33,43]. Kinetochore recruitment of CENP-E by BubR1 is dependent on a short helix present in the far C terminus of BubR1, a feature reminiscent of the interaction between Bub1 and CENP-F [43,44]. Early work in *Xenopus* egg extracts reported that CENP-E activates the checkpoint activity of BubR1 at unattached kinetochores and contributes to checkpoint silencing by stabilizing kinetochore-microtubule attachments [37,45–48]. However, subsequent work in human cells and Drosophila established that depletion or inhibition of CENP-E (and in Drosophila, CENP-*meta*) activity causes chromosome misalignment and induces a robust mitotic arrest [26,34,49]. Depletion of BubR1 in DLD-1 cells significantly reduces CENP-E at kinetochores [50], while other studies in high nocodazole show that depletion of BubR1 only mildly reduces CENP-E recruitment to unattached kinetochores [33,51]. Recent work has addressed the discrepancies, demonstrating that BubR1 is the major kinetochore recruiter of CENP-E during the maintenance of chromosome alignment and spindle checkpoint activation [43]. Upon prolonged nocodazole treatment, CENP-E can accumulate at kinetochores independently of BubR1 [43]. Thus, BubR1 is responsible for the initial and rapid recruitment of CENP-E to kinetochores, while a distinct pathway recruits CENP-E at unattached kinetochores [33,43,50,51]. The molecular basis for the alternative recruitment of CENP-E to kinetochores is not known.

Centromeric Protein F (CENP-F) was also identified as an interactor of CENP-E during the initial Y2H screen for CENP-E kinetochore binding partners [35]. In addition to mechanical roles at the nuclear envelope and mitochondrial outer membrane in G2 [52,53], CENP-F is recruited to outer kinetochores through a direct interaction with Bub1 in mitosis, providing a potential Bub1-dependent pathway for CENP-E recruitment to kinetochores [33,35,50,52]. Individual depletion of CENP-E and CENP-F indicate they show interdependency in their kinetochore localization [50,54,55]. Yet, in nocodazole-treated cells CENP-E is retained strongly at the kinetochore in the absence of CENP-F, indicating that CENP-F is not essential for CENP-E targeting to unattached kinetochores [33,54,55]. Whether CENP-E and CENP-F interact at the kinetochore remains controversial and a direct interaction between CENP-E and CENP-F has yet to be reconstituted *in vitro*. Both proteins localize to the fibrous corona and have been implicated in facilitating microtubule capture. However, only CENP-E is part of the outer kinetochore module that can be detached from the kinetochore after CDK1 inhibition. It notably colocalizes with the RZZ complex, Mad1 and the Dynein cargo adaptor Spindly in these detachable modules [12,13]. The outer corona is disassembled by Dynein upon kinetochore-microtubule attachment [16]. CENP-E is indeed removed from kinetochores in a Dynein-dependent manner [16,54], but the physical linkage between Dynein and CENP-E remains unknown.

Several other kinetochore components have been reported to interact with CENP-E and contribute to its kinetochore localization, including Nuf2, SKAP and Mad1; however whether they are direct interactions is unclear [24,51,56–59]. Some of these interactions may be facilitated by post-translational modifications (Figure 1). For instance, SUMO 2/3 modification of Nuf2 and NKAP promotes non-covalent interactions with the CENP-E kinetochore-binding domain [57,60]. Polyubiquitination of CENP-E by the Linear Ubiquitin Chain Assembly Complex (LUBAC) facilitates the recruitment CENP-E to attached kinetochores via an interaction with KNL-1 [61]. Similarly to CENP-F and Spindly, CENP-E is modified by prenylation of the C terminus by Farnesyl Transferase, to regulate the microtubule affinity and kinetochore localization of CENP-E [62–65]. Thus, kinetochore targeting of CENP-E is a tightly regulated process involving at least two redundant pathways to ensure the loading of CENP-E to kinetochores. The molecular basis for the BubR1-mediated recruitment of CENP-E during spindle activation is now well established. Future work is required to dissect the BubR1-independent pathway recruiting CENP-E to unattached kinetochores.

## CENP-E cooperates with other motors and microtubule tracks in lateral transport of chromosomes

**Figure 2:**
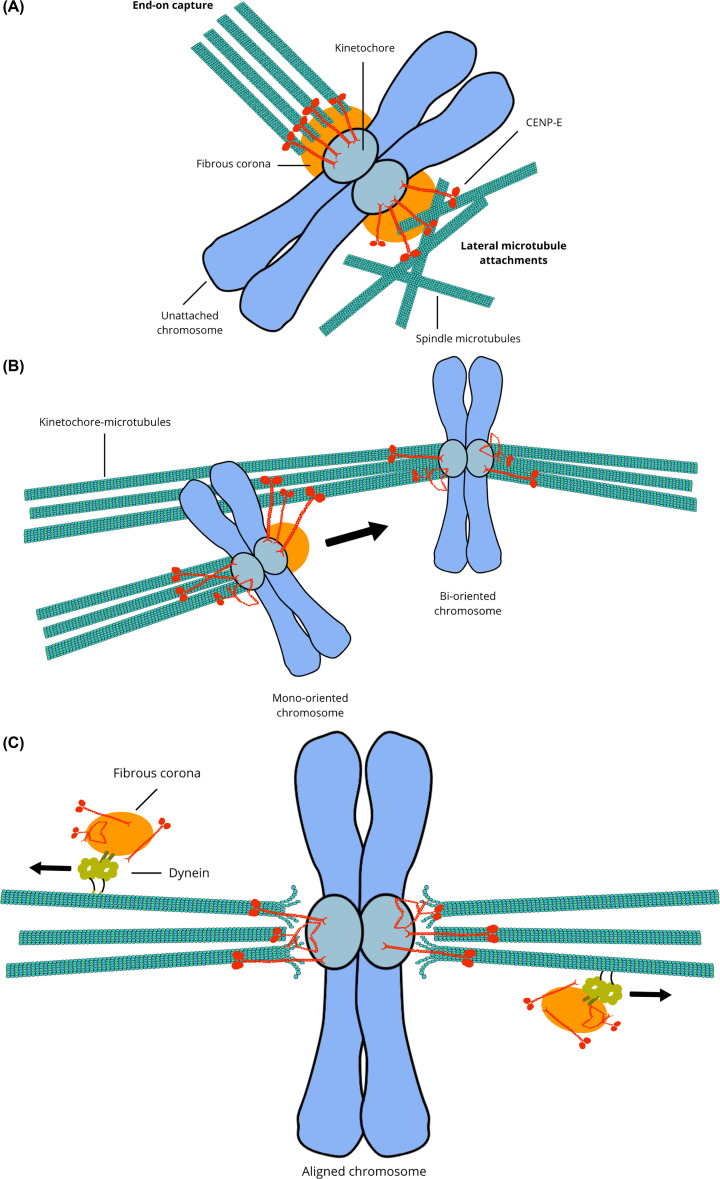
Roles of CENP-E at the kinetochore (**A**) The fibrous corona (orange) expands out from unattached kinetochores to facilitate interactions with microtubules. CENP-E is a major constituent of the expandable and detachable corona alongside RZZ, Mad1/2 and Spindly (not depicted). CENP-E motor domains laterally capture microtubules during the search and capture process (bottom-right). Plus-end directed activity promotes end-on capture by kinetochores (top-left). (**B**) CENP-E transports mono-oriented chromosomes to the equator laterally along neighbouring microtubules, guided by a preference for detyrosinated tubulin. (**C**) The fibrous corona (orange) is disassembled by Dynein (yellow) that transports CENP-E and other corona constituents to the spindle poles. A reduced pool of CENP-E is retained at kinetochores and helps maintain kinetochore attachments to dynamic microtubule ends at aligned chromosomes.

**Figure 3:**
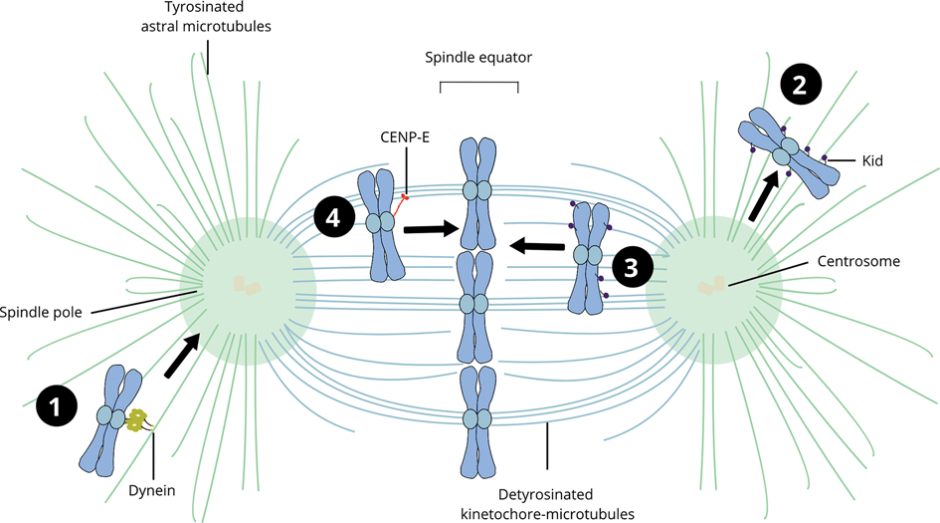
Motor-dependent chromosome congression Chromosomes congress to the equator prior to bi-orientation through a microtubule motor-dependent mechanism. Dynein is responsible for poleward transport of chromosomes that fall outside the interpolar region (**1**), preferentially along tyrosinated microtubules (green). Chromokinesin Kid (purple) generates forces along chromosome arms, which propel chromosomes outwards from the poles in random directions (**2**), but also contribute to stabilization of kinetochore-microtubule attachments and chromosome congression (**3**). CENP-E laterally transports unattached and mono-oriented chromosomes towards the spindle equator (**4**). The plus-end directed motor activity of CENP-E at the leading kinetochore powers chromosome transport along detyrosinated microtubules (blue).

Deciphering the contribution of CENP-E towards chromosome alignment has proved challenging, as perturbation of its motor activity by siRNA depletion results in chromosome misalignment and a prometaphase-like arrest, limiting further dissection of its activity [26,34,37,66–68]. A pioneering study by Kapoor et al. highlighted that chromosomes were able to align at the spindle equator prior to their bi-orientation in a CENP-E dependent manner, identifying a novel mechanism of chromosome congression independent of microtubule pulling forces (Figure 2B) [21]. The discovery of a CENP-E allosteric inhibitor, GSK923295A, that blocks the ATPase activity of the motor domain and recapitulates the CENP-E depletion phenotype also enabled rapid and acute inactivation of the motor to probe its activity in cells [69]. Using this inhibitor, several studies have demonstrated that CENP-E motility contributes to conversion of lateral to end-on kinetochore-microtubule attachments, chromosome congression and maintenance of alignment (Figure 2A–C) [23,70–72]. Motor co-depletion and inhibition further dissected the transport of mono-oriented chromosomes to the equator [23]. Congression of polar chromosomes is dependent on the synergistic actions of chromokinesins Kif4A and Kid, Dynein and CENP-E (Figure 3) [23,24,73]. Chromosomes successfully congress to the equator in the absence of arm-associated forces, also known as polar ejection forces (PEF), generated by chromokinesins [74]. However, the chromokinesin Kid plays an important role in chromosome congression in the absence of CENP-E [74–76]. Kinetochore Dynein counteracts the forces generated by Kid, laterally transporting mono-oriented chromosomes that lie outside the interpolar region back towards the spindle poles along tyrosinated astral microtubules (Figure 3) [23,24,77–79]. Following the focusing of mono-oriented chromosomes to spindle poles by Dynein, chromosomes are laterally transported by CENP-E to the equator (Figure 2B) [21,23,28,80]. CENP-E transports chromosomes along the detyrosinated spindle microtubules, which are favoured over tyrosinated astral microtubules (Figure 3) [23,25,28,80]. CENP-E also prefers detyrosinated microtubules *in vitro*, which promote longer run lengths and reduce the frequency of microtubule detachment when under load [80]. Chromosome alignment is established through the synergy between chromokinesins, Dynein and CENP-E motors, as well as the microtubule tracks.

## CENP-E plus-end directed motility is required for chromosome congression

*In vitro*, CENP-E was initially associated with minus-end directed motility despite the presence of a canonical kinesin motor domain at the N terminus [27,81]. Full-length CENP-E dimers purified from synchronized HeLa cells were unable to display microtubule gliding activity, only retaining the capacity to bind and pivot microtubules [30]. However, the crystal structure of the human CENP-E motor domain highlighted a number of structural similarities to plus end-directed kinesins KHC and Eg5, adopting a footprint on microtubules similar to kinesin-1 [82,83]. Subsequent work conclusively demonstrated that truncated *Xenopus* and human CENP-E motor domain possess robust plus-end directed microtubule gliding activity [28,67,84]. More recent reconstitutions with both truncated and full-length *Xenopus* CENP-E indicate the motor possesses a velocity of 20 µm/min with run lengths approximating 1.5–2.5 µm [28,29,68,71,80,84–87]. To date, processive motility has not been displayed for human CENP-E. Interestingly, the run length of CENP-E appears to be independent of the additional microtubule-binding site present in the C-terminal tail, which typically increases the processivity of kinesins [71,88–90]. Differences in buffer composition likely account for the variability in recorded motor velocities that range between 0.48 and 20 µm/min and for the discontinuity in its plus-end directed motion [28,68,71,84,85,91]. In addition to the inherent microtubule affinity of the kinesin motor domain, a short stretch of basic residues located in close proximity to the neck linker enhance microtubule affinity via one-dimensional diffusive motion [28,68,86,92]. In cells, CENP-E is tethered to kinetochores to transport chromosomes laterally along microtubules. Forces at kinetochores of around 700 pN are estimated to be necessary to move chromosomes [93]. Biophysical studies on CENP-E using optical trap microscopy showed that CENP-E dimers are capable of bearing loads up to 6 pN before stalling, comparable to the load-bearing capacity of kinesin-1, which supports its function in moving chromosomes [85]. CENP-E carries larger loads on detyrosinated microtubules, found in the spindle, when compared with tyrosinated microtubules [80]. Under load when bound to beads, CENP-E adopts a compact conformation of 25 nm, despite exhibiting an elongated structure during hydrodynamic analysis [28,94]. *In vivo*, fluorescence separation measurements indicate that CENP-E adopts a folded rather than an extended conformation, as both the motor and C-terminal tail appear to be positioned in close proximity to Hec1 at aligned kinetochores [95,96]. These data indicate functional CENP-E has a compact conformation with the stalk folding on itself, rather than an extended structure observed by rotary shadowing *in vitro* [28,94]. The contribution of plus-end directed motility of CENP-E to chromosome congression is now well-established. Evidence also suggests that active CENP-E adopts a compact functional conformation in mitosis.

## CENP-E properties to couple kinetochores to dynamic microtubules

Early studies showed microtubule depolymerization-dependent movement of chromosomes *in vitro* are blocked upon treatment with an inhibitory CENP-E antibody [97]. These data raised the question of whether the motor has a role in sustaining stable attachments to dynamic microtubule plus ends at aligned or segregating chromosomes. Several components of the outer kinetochore enable load-bearing microtubule attachments by processively tracking the growing and shrinking ends, including CENP-F, Ndc80 complex and Ska1 complex [53,98–102]. CENP-E also tip-tracks depolymerizing microtubules and stabilizes kinetochore-microtubule attachments [71,97,103,104]. In contrast with the enrichment of CENP-E at unattached kinetochores, only a residual amount of CENP-E is maintained at the kinetochores of aligned chromosomes following Dynein-dependent stripping of the fibrous corona (Figure 2C) [16,32,54]. The remaining pool of CENP-E at bi-oriented kinetochores is essential for the maintenance of chromosome alignment: inhibition of CENP-E ATPase activity results in the poleward movement of chromosomes from the metaphase plate [71]. *In vitro*, full-length CENP-E is able to processively track both growing and shrinking microtubules upon reaching the dynamic plus ends, indicating that CENP-E may contribute to maintaining load-bearing microtubule attachments to kinetochores (Figure 2C) [71]. Plus-end tracking of dynamic microtubules is dependent on the additional microtubule-binding site located in the far C terminus [71]. This second microtubule-binding region has a nanomolar affinity for microtubules and binds electrostatically to the microtubule lattice, utilizing fast diffusion along the lattice to prevent motor dissociation once it reaches the tip [71,105]. In contrast with kinesin-8 and kinesin-13 motors, which also associate with microtubule tips in mitosis to control their dynamics, CENP-E does not modulate microtubule dynamics *in vitro* [71,88,89,106]*.* Therefore, it is unlikely to directly regulate microtubule dynamics at the interface of the outer kinetochore. However, CENP-E has been previously shown to recruit CLASP 1 and 2 (Xorbit in *Xenopus*) *in vivo*, to promote microtubule turnover at the kinetochore [107–109]. Co-immunoprecipitation experiments indicate that a non-motor region of CENP-E recruits the C terminus of CLASP to kinetochores but whether the interaction is direct is not known [108,109]. Overall, *in vitro* reconstitutions have thus provided key insights into the motile and biochemical properties of CENP-E to maintain stable-kinetochore microtubule attachments.

## Regulation of CENP-E motor activity

The activity of CENP-E at the kinetochore is tightly regulated. In a similar fashion to Kinesin-1 and Kinesin-3, CENP-E is proposed to self-regulate its microtubule motor activity [110–112]. CENP-E primarily adopts an elongated structure in solution. Rotary shadowing shows that a single CENP-E dimer spans an extended length up to 230 nm, adopting a variety of flexible conformations *in vitro* [28]. The high degree of flexibility in the CENP-E stalk may facilitate autoinhibitory interactions between the motor and the C-terminal tail [29]. Recent work has shown that attachment of full-length CENP-E to beads *in vitro* increases the activity of the motor, indicating a cargo-induced activation mechanism may relieve the motor from autoinhibition [71,87]. However, whether adaptor proteins *in vivo* are responsible for regulating CENP-E activity by such a mechanism is unknown. One study proposed that the C terminus of *Xenopus* CENP-E interacts with the motor domains of CENP-E, although this interaction could only be detected by surface plasmon resonance (SPR) [29]. Incubation of the recombinantly purified CENP-E C terminus with the truncated motor domain resulted in dose-dependent inhibition of microtubule gliding activity, an effect that could be relieved by Cdk1/Mps1 phosphorylation [29]. Given the C-terminal tail also binds to microtubules, it is not clear whether it prevents the motor from stepping through steric hindrance or inhibits the motor directly from these studies. The stalk also has a specific role in regulating CENP-E activity [87,94]. Artificial shortening of the CENP-E stalk and exchange for the rigid coiled-coils of kinesin-1 results in a CENP-E motor that cannot rescue chromosome misalignments *in vivo.* However, we cannot rule out the engineered constructs are defective independently of the potential regulatory function of the coiled coil regions [87,96]. Whether removal of the stalk favours autoinhibitory interactions between the motor head and tail, impacts the load bearing capacity of the motor or perturbs protein–protein interactions is currently unclear. However, it highlights that the length and flexibility of the stalk is important for CENP-E activity.

Phosphorylation regulates CENP-E motor activity and the end-on capture of microtubules [68]. Multiple Cdk1 and Aurora A/B kinase consensus phosphorylation sites are present along the length of CENP-E, many of which are uncharacterized [68,113]. Aurora A/B kinases phosphorylate CENP-E on threonine 422, a residue that overlaps into a highly conserved PP1 docking site known as the RVXF motif and in turn disfavours the direct interaction between CENP-E and PP1 when phosphorylated [68]. Injection of a T422 phospho-specific CENP-E antibody into human cells and mutagenesis studies demonstrated that T422 phosphorylation is required to promote CENP-E-dependent chromosome congression [68]. The second microtubule-binding site in the C-terminal tail is also phosphorylated [113]. Upon identification of the C-terminal microtubule-binding site in human CENP-E, Liao et al. reported that Cdk1 phosphorylation of this disordered tail reduced its microtubule affinity [114]. In contrast, treatment of the *Xenopus* CENP-E tail with Mps1 or Cdk1 had no effect on microtubule binding affinity [29]. Unfortunately, neither study reported the specific phosphorylation sites targeted by Cdk1 and Mps1 *in vitro* [29,114]. Recent work has suggested that phosphorylation of serine 2613 promotes end-on microtubule capture and tip-tracking activity of the CENP-E tail, but whether this phosphorylation event is catalysed by BubR1 as hypothesized by Huang et al. or via a distinct mitotic kinase in mammalian cells remains to be established [39,40]. Other phosphorylation sites have been identified in phosphoproteomic screens. [115,116]. Nine identified sites on CENP-E have been mutated but did not reveal any mitotic phenotype indicating they are not likely to be major regulatory sites [68]. CENP-E is also post-translationally modified through farnesylation and ubiquitination to regulate its function and kinetochore-targeting, although the molecular basis is not known [61,62]. In summary, CENP-E activity is regulated by cargo binding and post-translational modifications, but the mechanisms underlying CENP-E function in the context of chromosome alignment and segregation is yet to be defined.

## Concluding remarks

CENP-E plays a critical role in mammalian chromosome alignment. Recent work has elucidated how CENP-E loads onto kinetochores through BubR1, yet highlighted that in the absence of BubR1, additional BubR1-independent pathways could recruit CENP-E to kinetochores albeit with different kinetics [43]. *In vitro* work with *Xenopus* CENP-E has given us mechanistic insights into CENP-E as a molecular machine that moves chromosomes and maintains attachments to dynamic microtubule ends. However, *Xenopus* CENP-E is constitutively active while the activity of full-length human CENP-E has not been demonstrated so far [30], indicating their sequence divergence may underlie different activation and regulatory mechanisms. Outstanding questions such as how CENP-E is recruited to kinetochores independently of BubR1, how CENP-E is modulated by its cargos and how CENP-E molecules cooperate with each other and with other motors at kinetochores to move chromosomes remain to be answered.

## Summary

- CENP-E is a kinesin-7 kinetochore-targeted motor that walks to microtubule plus-ends.
- Mono-oriented polar chromosomes require a CENP-E-dependent mechanism of congression.
- BubR1 targets CENP-E to kinetochores, but other pathways are also responsible for CENP-E kinetochore loading.
- Detyrosination of spindle microtubules guides lateral chromosome transport to the equator by CENP-E.
- CENP-E activity is regulated by post-translational modifications, protein interactions and autoinhibition.

## Open Access

Open access for this article was enabled by the participation of University of Edinburgh in an all-inclusive *Read & Publish* pilot with Portland Press and the Biochemical Society under a transformative agreement with JISC.

## Abbreviations

CCAN: centromeric associated network of proteins
CENP-E: centromere protein E
PEF: polar ejection forces
RZZ: Rod-Zw10-Zwilch
